# Project DEAL — Germany is leading the way in the transition to open access publishing

**DOI:** 10.1002/acm2.12890

**Published:** 2020-04-30

**Authors:** Michael Mills

## 1 Introduction

Pioneers in the open access movement are gratified, and more than a little relieved, to see the world moving in our direction. Over the past several years, several consortiums of national research institutions and libraries, notably Sweden, the Netherlands, Norway, Hungary, and Germany, have initiated negotiations with the leading electronic journal publishing houses including Wiley, Springer, and Elsevier. This editorial focuses on one of the more successful initiatives, Project DEAL (Projekt DEAL), in Germany. The goal of this endeavor is to rewrite the nationwide agreements for licensing content in electronic journals. Under the terms of these negotiations, scientists in Germany would get immediate and full online access to most of the electronic content of a publisher’s journals. In exchange, the publication costs for the German authors would be subsidized, and all of their articles would be published immediately open access. This “publish and read” model makes all articles available to anyone with web access, and additionally, authors have access to all electronic journals of the publisher, including archives.

On January 15, 2019, the DEAL consortium found a willing publisher in Wiley, and signed a 3‐year contract to bring a published and read model to libraries and research institutions in Germany. The Wiley deal is expected to cover and fund an anticipated 9,500 articles per year. https://www.the-scientist.com/news-opinion/project-deal-in-germany-reaches-agreement-with-springer-nature-66342. Archive access extends back to 1997 for all electronic Wiley journals. The cost to publish an article open access is approximately $3,050 US, which is paid centrally by the academic institutions responsible for the article. https://authorservices.wiley.com/author-resources/Journal-Authors/open-access/affiliation-policies-payments/german-projekt-deal-agreement.html

“On a practical level, researchers in Germany will find publishing their papers open access much simpler, says Frank Sander, head of the Max Planck Digital Library Services (MPDL Services), a company in Munich, Germany, formed to manage the Project DEAL contracts. Instead of researchers paying open‐access fees to a journal covered by the agreement, MPDL Services will pay. MPDL Services will then bill the authors’ institution. This arrangement, which was set up to support the earlier Project Deal agreement with Wiley, means German funding for publications will shift from libraries to research institutions, he notes.” https://www.sciencemag.org/news/2019/08/more-700-german-research-institutions-strike-open-access-deal-springer-nature

“With Wiley, we found a publisher on the other side of the table that was willing to make this transition [to open access] in partnership with us,” says Gerard Meijer, a molecular physicist at the Fritz Haber Institute of the Max Planck Society and a member of the DEAL negotiations team. “We consider the contract with Wiley as a blueprint for the contracts that are going to follow.”

“We believe [these deals are] the right thing to do on behalf of our community,” says Judy Verses, the executive vice president of research at Wiley. “Researchers in our community have made clear that open research and open access is a critical part of the future.” According to Verses, Wiley is currently in active discussions for new licensing deals with consortia in other countries, including in the US. https://www.the-scientist.com/news-opinion/as-elsevier-falters--wiley-succeeds-in-open-access-deal-making-65664

To be eligible:

- You must publish open access in one of Wiley's fully open access journals (such as *JACMP*) or a subscription journal that offers OnlineOpen (hybrid journal) (such as *Medical Physics*)
- You must be the responsible corresponding author and affiliated with a publicly or privately funded German research institution entitled under Projekt DEAL at the point of acceptance
- The affiliation you select during submission must be stated on the published paper
- All primary research and review articles qualify
- Wiley Open Access Accounts cannot be used to cover additional charges (e.g., cover, color, and page charges), which individual journals administer separately.

https://authorservices.wiley.com/author-resources/Journal-Authors/open-access/affiliation-policies-payments/german-projekt-deal-agreement.html

The Open Access Scholarly Publishers Association has some reservations, which they discuss in the context of Plan S, but which applies equally to Project DEAL. (See my previous editorial on Plan S in the JACMP March 2019 issue.)

OASPA’s main concern relating to Plan S, however, is that discussions and solutions continue to be focused on the largest, mixed‐model publishers. While it is this segment of the market on which funders’ attention — and spend — is concentrated, the vast majority of publishers within the so‐called “Long Tail” (the majority of OASPA’s members) appear to be absent from the focus of Plan S. Many of these publishers are too small to negotiate the kind of “transformative” national Big Deals we are seeing for the largest publishers, while exclusively open access publishers without legacy subscription businesses are also unable to participate. Many are not even of sufficient size to make agreements directly with institutions. https://oaspa.org/oaspa-feedback-on-plan-s-implementation-guidance/

What does this mean for the *JACMP*? It is likely that consortiums such as Project Deal will continue to flourish and become a larger part of the landscape. This will continue to put pressure on the print/subscription/print advertising business model of traditional journals. Published by a world’s leading publisher, the *JACMP* is well positioned to take advantage of such consortium deals in the short term. We may see large library systems in the US, such as the Universities of California, Texas, New York, and Florida show interest in such deals in the near future. There may even be a national US initiative, although it is not certain how that would emerge. Such deals have the potential of increasing interest in open access publishing, both because the authors would no longer be responsible for the article publication charge (APC), but also because such sweeping deals would bring higher revenue to journals with an APC lower than the negotiated cost per article. Both would benefit the *JACMP*.

The idea of a clinical medical physics journal that was published open access and successful first presented itself to me in November of 1997, when I was 45. I am finishing this editorial on my 68th birthday. I am grateful to have lived to see the day!

Finally, please view this twitter link for the JACMP 20th Anniversary Social Media Campaign: https://twitter.com/hashtag/JACMP20?src=hashtag_click&f=live

